# Single-distance and dual-slope frequency-domain near-infrared spectroscopy to assess skeletal muscle hemodynamics

**DOI:** 10.1117/1.JBO.28.12.125004

**Published:** 2023-12-14

**Authors:** Cristianne Fernandez, Giles Blaney, Jodee Frias, Fatemeh Tavakoli, Angelo Sassaroli, Sergio Fantini

**Affiliations:** Tufts University, Department of Biomedical Engineering, Medford, Massachusetts, United States

**Keywords:** diffuse optics, near-infrared spectroscopy, frequency-domain, skeletal muscle, blood flow, oxygen consumption

## Abstract

**Significance:**

Non-invasive optical measurements of deep tissue (e.g., muscle) need to take into account confounding contributions from baseline and dynamic optical properties of superficial tissue (adipose tissue).

**Aim:**

Discriminate superficial and deep tissue hemodynamics using data collected with frequency-domain (FD) near-infrared spectroscopy (NIRS) in a dual-slope (DS) configuration.

**Approach:**

Experimental data were collected *in vivo* on the forearm of three human subjects during a 3-min arterial occlusion or 1-min venous occlusion. Theoretical data were generated using diffusion theory for two-layered media with varying values of the reduced scattering coefficient ($\mu_{s}^{\prime }$) (range: 0.5 to $1.1\;{\mathrm{mm}}^{-1}$) and absorption coefficient ($\mu_{a}$) (range: $0.005-0.015\;{\mathrm{mm}}^{-1}$) of the two layers, and top layer thickness (range: 2 to 8 mm). Data were analyzed using diffusion theory for a homogeneous semi-infinite medium.

**Results:**

Experimental data *in vivo* were consistent with simulated data for a two-layered medium with a larger $\mu_{s}^{\prime }$ in the top layer, comparable absorption changes in the top and bottom layers during venous occlusion, and smaller absorption changes in the top vs. bottom layers during arterial occlusion.

**Conclusions:**

The dataset generated by DS FD-NIRS may allow for discrimination of superficial and deep absorption changes in two-layered media, thus lending itself to individual measurements of hemodynamics in adipose and muscle tissue.

## 1 Introduction

Near-infrared spectroscopy (NIRS) is a non-invasive optical technique that has been applied to measurements of the perfusion and oxygenation of various biological tissues, including skeletal muscle^1^^,^^2^ and brain.^3^ NIRS studies have employed continuous-wave (CW),^4^^–^^7^ time-domain (TD),^8^ and frequency-domain (FD)^9^^,^^10^ systems, which are based on constant, pulsed, and intensity-modulated illumination, respectively. To achieve high accuracy and reliability, non-invasive NIRS measurements of skeletal muscle and brain must take into account potentially confounding contributions to the optical signals from superficial tissue, namely skin and adipose tissue in the case of muscle measurements^2^ or scalp and skull tissue in the case of brain measurements.^11^

Focusing on muscle studies, it was observed that NIRS data need to be corrected for the adipose tissue thickness (ATT) to yield measurements that are representative of muscle tissue.^6^^,^^12^^,^^13^ To this aim, TD-NIRS data were used either to generate correction curves derived from the ratio of partial optical pathlength in muscle to the total optical pathlength,^8^ or to yield separate measurements for the top layer (adipose tissue) and bottom layer (muscle) by modeling the tissue as a two-layered medium.^14^ CW-NIRS for skeletal muscle measurements has been commonly implemented by placing a single illumination point (source) and a single optical collection point (detector) on the tissue surface. This arrangement is commonly referred to as single-distance (SD) since it relies on a single source–detector separation. In this approach, the measured quantity is the optical intensity (I) as well as its changes associated with muscle hemodynamics and deoxygenation (ΔI).^6^^,^^12^ These SD intensity (SDI) measurements are known to have a greater sensitivity to superficial tissue compared to single distance phase (SDϕ) measurements obtained with FD-NIRS.^15^^,^^16^ In the case of FD-NIRS, I and ϕ represent the amplitude and the phase, respectively, of the photon-density waves that result from modulated illumination.

One alternative method that has been applied to the brain,^15^^–^^17^ and skeletal muscle^18^ is the dual-slope (DS) approach. The DS approach aims to measure the changes in the slope of either the linearized intensity ($\ln(\rho^{2}I)$) or phase (ϕ) as a function of source–detector distance (ρ), without any need of calibration for source emission or detector sensitivity factors. This is done by using a special arrangement of two sources and two detectors on the tissue surface, and by averaging the two paired single-slopes that are measured using each single source in combination with the two detectors.^19^ We have shown that in comparison with SD methods, the DS approach offers preferential sensitivity to deeper localized absorption changes in otherwise homogeneous media,^19^ and that hemodynamics measured on the human head are more closely representative of cerebral tissue than superficial scalp/skull tissue.^16^^,^^20^ In this work, we explore the ability of DS FD-NIRS measurements in limiting or assessing the contribution of adipose tissue to the NIRS measurements of skeletal muscle.

Two commonly used NIRS protocols to measure blood flow and oxygen consumption in skeletal muscle involve the use of a venous occlusion and an arterial occlusion, respectively. These protocols cause hemodynamic and oxygenation changes in limb tissues (skeletal muscle, skin, adipose tissue, etc.) that are distal to the pneumatic cuff used to perform the vascular occlusion. A venous occlusion is achieved by inflating a pneumatic cuff that is wrapped around the limb to a pressure of 40 to 60 mmHg, which is above venous pressure, effectively causing the accumulation of blood in distal tissues. Such protocols have been used to measure muscle blood flow from the initial rate of accumulation of blood following the onset of the occlusion.^2^^,^^7^^,^^21^ Previous work has shown that blood flow in adipose tissue is greater than in skeletal muscle at rest,^22^ as confirmed by NIRS measurements on a population of 78 subjects with ATT ranging from 1.5 to 9 mm.^6^ An arterial occlusion is achieved similarly but with a greater cuff inflation pressure of about ∼200  mm Hg or more, which is above the systolic pressure, effectively blocking both the inflow and outflow of blood to the measured tissue.^23^ During an arterial occlusion, the oxygen consumption is measured through the rate of deoxygenation of heme compounds [hemoglobin (Hb) and myoglobin (Mb)] in muscle. Because the near-infrared absorption spectra of hemoglobin and myoglobin have extremely similar shapes, being both dominated by their heme group(s), NIRS methods can only measure the combined concentrations of hemoglobin and myoglobin in their oxygenated state (oxy[Hb + Mb]) or deoxygenated state (deoxy[Hb + Mb]). In this article, we use the compact notation of O and D for oxy[Hb+Mb] and deoxy[Hb+Mb], respectively, and T for the total heme concentration (T=O+D). In the absence of blood redistribution within the muscle, the oxygen desaturation of heme groups results in equal rates of decrease of O and increase of D. Adipose tissue has been shown to have a lower oxygen consumption than skeletal muscle at rest.^6^^,^^22^^,^^24^ The different hemodynamic and deoxygenation behavior of adipose tissue and muscle tissue during a venous or arterial occlusion render these protocols useful tools to investigate the relative sensitivity of NIRS measurements to adipose and muscle tissues.

Here we use the DS method with both I and ϕ measurements on the human forearm during either an arterial or venous occlusion. For each occlusion protocol, we report results on three subjects. We note that DS measurements comprise two sets of SD data collected at two different source–detector separations (25 and 35 mm in this work). The main objective of this work is to investigate the information content of different data types (namely SDI, SDϕ, DSI, and DSϕ) in relation to the in-homogeneous hemodynamics in superficial tissue (skin and adipose layer) and deeper tissue (skeletal muscle) in response to vascular occlusions. To guide our interpretation of the experiments *in vivo*, we also performed simulations in two-layered media to simulate the effects of different baseline tissue properties, different absorption changes in the two layers, and different top layer thicknesses on the measured optical data generated by FD-NIRS in both SD and DS configurations.

## 2 Methods

### 2.1 Experimental Protocol and Instrumentation

**Fig. 1:**
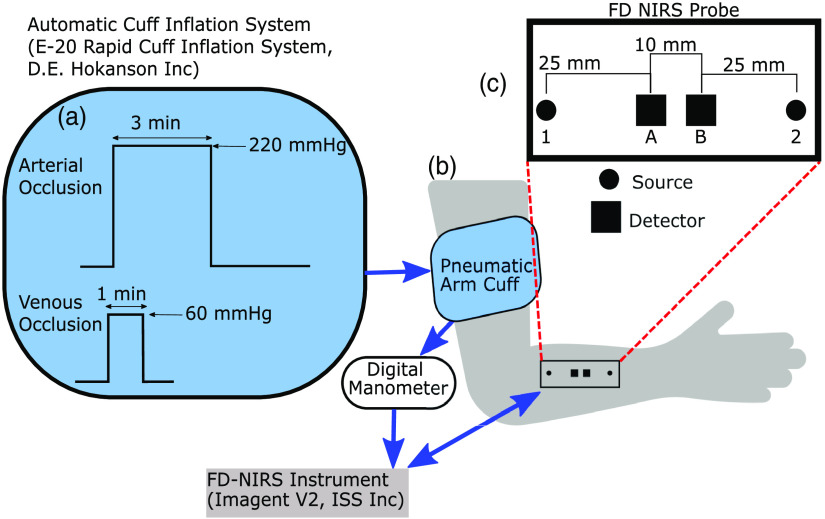
Experimental setup for frequency-domain (FD) near-infrared spectroscopy (NIRS) measurements on the human forearm during an arterial occlusion or venous occlusion. (a) The timing of the occlusions achieved by inflation of a pneumatic arm cuff using an automatic cuff inflation system [E-20 Rapid Cuff Inflation System, D.E. Hokanson, Inc. (Bellevue, Washington, United States)]. (b) A pneumatic arm cuff [SC12D, D.E. Hokanson, Inc. (Bellevue, Washington, United States)] was placed on the subject’s upper arm. The cuff pressure was measured continuously using a Series 626 Pressure Transmitter Dwyer Instruments Inc. (Michigan City, Indiana, United States) and recorded synchronously with the data collected by the FD-NIRS system [ISS Imagent V2 (Champaign, Illinois, United States)]. An optical probe connected to the FD-NIRS system was placed on the forearm above the brachioradialis muscle. (c) Schematic of the FD-NIRS optical probe with numbers used to label optical sources and letters to label detectors. This arrangement allowed for two single-distance (SD) measurements at a source–detector separation of 25 mm (1A and 2B), two SD measurements at a source–detector separation of 35 mm (1B and 2A), and one dual-slope measurement based on data collected by all combinations of sources and detectors (1AB2).

A total of 4 subjects [3 female (F), 1 male (M); age range 22 to 29 years] participated in a study approved by the Tufts University Institutional Review Board (IRB) for FD-NIRS measurements conducted on the human forearm. Two experimental protocols, one involving a venous occlusion and one involving an arterial occlusion, are schematically illustrated in Fig. 1(a). The venous occlusion protocol consisted of a 1 min baseline, followed by a 1 min venous occlusion achieved by inflating a pneumatic cuff wrapped around the upper arm to a pressure of 60 mmHg and a final 1 min recovery. The arterial occlusion protocol consisted of a 1 min baseline followed by a 3 min arterial occlusion achieved by inflating a pneumatic cuff wrapped around the upper arm to a pressure of 220 mmHg and a final 3 min recovery. Subjects 2 and 4 participated in both protocols, whereas subject 1 elected to only participate in the arterial occlusion protocol and subject 3 only in the venous occlusion protocol, as listed in Table 1. Therefore, this work reports results on three subjects for each occlusion period.

**Table 1:** Summary of the baseline measurements for each subject (sub) before the arterial occlusion and venous occlusion protocols (prot). Adipose tissue thickness (ATT), mean ± standard deviation of the absorption coefficient ($\mu_{a}$) and reduced scattering coefficient ($\mu_{s}^{\prime }$) during the baseline period at the two wavelengths of 690 and 830 nm, and generalized mean optical pathlength for intensity (I) (${\left\langle L\right\rangle }_{I}$) and phase (ϕ) (${\left\langle L\right\rangle }_{\phi }$). ${\left\langle L\right\rangle }_{I}$ and ${\left\langle L\right\rangle }_{\phi }$ are reported for both wavelengths and source–detector distances (ρ): $\rho_{1}=25\;\mathrm{mm}$ and $\rho_{2}=35\;\mathrm{mm}$.

Prot			$\mu_{a}$ (${\mathrm{mm}}^{-1}$)		$\mu_{s}^{\prime }$ (${\mathrm{mm}}^{-1}$)		${\left\langle L\right\rangle }_{I}$ (mm)				${\left\langle L\right\rangle }_{\phi }$ (mm)			
	Sub	ATT (mm)	690 nm	830 nm	690 nm	830 nm	690 nm	830 nm	690 nm	830 nm	690 nm	830 nm	690 nm	830 nm
							$\rho_{1}$	$\rho_{2}$	$\rho_{1}$	$\rho_{2}$	$\rho_{1}$	$\rho_{2}$	$\rho_{1}$	$\rho_{2}$
Arterial occlusion	1	2.0 ± 0.3	0.0172 ± 0.0007	0.0160 ± 0.0004	0.58 ± 0.01	0.56 ± 0.01	106	153	107	154	10.3	15.6	11.1	16.8
	2	3.4 ± 0.2	0.0161 ± 0.0002	0.0190 ± 0.0001	0.551 ± 0.003	0.477 ± 0.003	106	152	92.7	133	10.8	16.4	8.15	12.2
	4	6.0 ± 0.5	0.0198 ± 0.0001	0.01756 ± 0.00008	0.581 ± 0.003	0.596 ± 0.003	100	144	106	153	8.64	13.0	10.2	15.4
Venous occlusion	2	3.4 ± 0.2	0.0150 ± 0.0001	0.0177 ± 0.0001	0.560 ± 0.003	0.5140.003	109	158	98.6	142	12.0	18.2	9.26	13.9
	3	4.4 ± 0.2	0.0156 ± 0.0003	0.0185 ± 0.0003	0.547 ± 0.006	0.494 ± ±0.006	107	154	95.1	136	11.3	17.1	8.57	12.9
	4	6.0 ± 0.5	0.0185 ± 0.0004	0.01839 ± 0.00009	0.571 ± 0.002	0.598 ± 0.003	102	147	105	151	9.30	14.0	9.61	14.5

All subjects were placed in a seated position with the right forearm on a flat surface. Before starting the experiment, ATT was measured with a skinfold caliper at the measurement location and the ATT values are reported in Table 1 for each subject. First, a pneumatic arm cuff [SC12D, D.E. Hokanson, Inc. (Bellevue, Washington, United States)] was placed on the upper right arm [Fig. 1(b)]. The cuff pressure was controlled using an automatic cuff inflation system [E-20 Rapid Cuff Inflation System, D.E. Hokanson, Inc. (Bellevue, Washington, United States)] to inflate the cuff to the desired pressure. The cuff pressure was measured continuously using a digital manometer [Series 626 Pressure Transmitter Dwyer Instruments Inc. (Michigan City, Indiana, United States)], and the pressure data were recorded synchronously with the FD-NIRS data.

An optical probe for FD-NIRS measurements was placed on the forearm over the brachioradialis muscle and secured via black athletic bandaging to guarantee good contact with the skin. The optical probe contained two pairs of illumination optical fibers and two collection optical fiber bundles that were connected to the light sources and optical detectors, respectively, of a commercial ISS Imagent V2 (Champaign, Illinois, United States) (modulation frequency: 140.625 MHz). Each pair of illumination fibers was connected to two laser diodes that emitted light at 690 and 830 nm. The light sources were time-multiplexed, so only one source was on at any given time. The sampling rate for data collection ranged from ∼2 to 10 Hz, with lower sampling rates used to increase integration time for each source–detector pair to decrease phase noise. The two optical collection points in the optical probe are labeled A and B, whereas the two source locations (each featuring illumination at two wavelengths) are labeled 1 and 2 [Fig. 1(c)]. This arrangement allows for two SD measurements at a source–detector separation of 25 mm (1A and 2B), two SD measurements at a source–detector separation of 35 mm (1B and 2A), and one DS measurement (1AB2) that combines data collected by all source–detector combinations.

### 2.2 Theoretical Simulations

An analytical approach based on diffusion theory for a two-layer medium^25^^,^^26^ with an infinitely thick bottom layer was used to guide the interpretation of the experimental results from both occlusion protocols. The choice of an infinitely thick bottom layer is based on the fact that, for typical optical properties of tissue, all data types considered in this work have a sensitivity of at least 90% to the top 20 mm of tissue.^27^ Between the adipose tissue layer and the radius bone, one finds the brachioradialis muscle and the extensor carpi radialis, which have a combined thickness of about 18 mm in adults younger than 50 years old.^28^ Considering a superficial adipose tissue thickness >2  mm, the bone depth is at about 20 mm or more (depending on individual anatomy). We verified that for typical optical properties of tissue, all data types considered in this work have a sensitivity of at least 90% to the top 20 mm of tissue thus justifying our choice to perform simulations for two-layered media in which the bottom layer extends indefinitely. For each simulation, we defined specific values of the absorption coefficient ($\mu_{a}$) in the top and bottom layers ($\mu_{a,\text{top}}$ and $\mu_{a,\mathrm{bot}}$, respectively), the reduced scattering coefficient ($\mu_{s}^{\prime }$) in the top and bottom layers ($\mu_{s,\text{top}}^{\prime }$ and $\mu_{s,\mathrm{bot}}^{\prime }$, respectively), and the top layer thickness ($L_{\text{top}}$). This was considered as a simulation of the baseline conditions. We then computed the complex reflectance ($\tilde{R}$), whose amplitude (I) and phase (ϕ) represent the FD-NIRS signal, for each source–detector pair of the probe geometry in Fig. 1(c). This complex reflectance was used to obtain the effective optical properties (i.e., the optical properties of an effective semi-infinite homogeneous medium) at baseline. Absorption perturbations in the top and bottom layers ($\Delta \mu_{a,\text{top}}$ and $\Delta \mu_{a,\mathrm{bot}}$, respectively) were then introduced, and the associated change in $\tilde{R}$ ($\Delta \tilde{R}$) was calculated. Finally, the effective absorption coefficient change ($\Delta \mu_{a}$) associated with $\Delta \tilde{R}$ were obtained as described in Sec. 2.3. Processing of the experimental and simulated data was done in the same way to allow for a fair comparison.

To study the effect of different parameters of the two-layered medium ($L_{\text{top}}$ and optical properties of the two layers) on the recovered (effective) $\Delta \mu_{a}$, the top and bottom layer $\mu_{a}$, $\mu_{s}^{\prime }$, and $L_{\text{top}}$ were all varied. In all simulations, three cases of $\Delta \mu_{a,\mathrm{top}}$ and $\Delta \mu_{a,\mathrm{bot}}$ were considered: (1) $\Delta \mu_{a,\text{top}}<\Delta \mu_{a,\mathrm{bot}}$, (2) $\Delta \mu_{a,\text{top}}=\Delta \mu_{a,\mathrm{bot}}$, and (3) $\Delta \mu_{a,\text{top}}>\Delta \mu_{a,\mathrm{bot}}$. These cases were chosen to represent the different hemodynamic responses in the two layers during the two occlusions considered: lower oxygen consumption in superficial adipose tissue versus muscle tissue at rest (i.e., $\left|\Delta \mu_{a,\text{top}}|<|\Delta \mu_{a,\mathrm{bot}}\right|$ during an arterial occlusion) and greater blood flow in superficial adipose tissue vs. muscle tissue at rest (i.e., $\Delta \mu_{a,\text{top}}>\Delta \mu_{a,\mathrm{bot}}$ during a venous occlusion). The optical properties of the two layers at baseline were chosen so that the effective optical properties (defined in Sec. 2.3) were within the range of those measured experimentally (Table 1).

### 2.3 Determining Effective Optical Properties and Effective Absorption Changes

Methods described here were applied to both experimental and simulated reflectance ($\tilde{R}$) data. Effective optical properties, $\mu_{a}$ and $\mu_{s}^{\prime }$ (for each wavelength in the case of experimental data), were calculated using a multi-distance iterative recovery method^29^ based on a semi-infinite homogeneous medium with extrapolated boundary conditions. The optical probe was used in the self-calibrating (SC) configuration,^30^ which allows one to collect DS intensity (DSI) and DS phase (DSϕ) to yield effective $\mu_{a}$ and $\mu_{s}^{\prime }$. For the experimental data described in Sec. 2.1, effective $\mu_{a}$ and $\mu_{s}^{\prime }$ at baseline were taken as the average over time from the start of the experiment to 1 s before the start of cuff inflation. Table 1 reports the effective $\mu_{a}$ and $\mu_{s}^{\prime }$ for each subject, experimental protocol, and wavelength.

Changes in I or ϕ recorded over a time interval Δt at a single ρ were translated into associated effective absorption changes ($\Delta \mu_{a}$) as follows:

$$\Delta \mu_{a,M}(\rho ,\Delta t)=-\frac{\Delta M(\rho ,\Delta t)}{{\left\langle L\right\rangle }_{M}(\rho )},$$ (1)

where M is either $\ln(\rho^{2}I)$ or ϕ for SD data and ${\left\langle L\right\rangle }_{M}$ is the generalized mean optical pathlength associated with data type M (${\left\langle L\right\rangle }_{M}$ indeed has units of length if M is dimensionless, as it is the case here for both $\ln(\rho^{2}I)$ and ϕ (the latter being expressed in radians) [we note that a dimensionless constant factor in the argument of the logarithm for $M=\ln(\rho^{2}I)$ cancels out in ΔM]). The generalized mean optical pathlength is essentially the opposite of the partial derivative of the data type M [$\ln(\rho^{2}I)$ or ϕ] with respect to $\mu_{a}$ and was calculated using the effective $\mu_{a}$ and $\mu_{s}^{\prime }$ for each optical wavelength (λ) and ρ as reported in Table 1. Note that ${\left\langle L\right\rangle }_{M}/\rho$ is the conventional differential pathlength factor (DPF) in the case of I data. We averaged the $\Delta \mu_{a}$ calculated using SD data from the two different source–detector pairs that feature the same source–detector separation leading to a single value for ${\left\langle L\right\rangle }_{I}(\rho_{1})$, ${\left\langle L\right\rangle }_{I}(\rho_{2})$, ${\left\langle L\right\rangle }_{\phi }(\rho_{1})$, and ${\left\langle L\right\rangle }_{\phi }(\rho_{2})$, where $\rho_{1}=25\;\mathrm{mm}$ and $\rho_{2}=35\;\mathrm{mm}$. From these values of generalized pathlengths, we computed a differential slope factor for data type M (${\mathrm{DSF}}_{M}$) as follows:

$${\mathrm{DSF}}_{M}(\rho_{1},\rho_{2})=\frac{{\left\langle L\right\rangle }_{M}(\rho_{2})-{\left\langle L\right\rangle }_{M}(\rho_{1})}{\rho_{2}-\rho_{1}}.$$ (2)

Changes in DS data for data type M (ΔDSM) (i.e., changes in the slope of either $\ln(\rho^{2}I)$ or ϕ versus ρ) over time Δt were then converted into associated absorption changes as follows:

$$\Delta \mu_{a,\mathrm{DS}M}(\rho_{1},\rho_{2},\Delta t)=-\frac{\Delta \mathrm{DS}M(\rho_{1},\rho_{2},\Delta t)}{{\mathrm{DSF}}_{M}(\rho_{1},\rho_{2})}.$$ (3)

Considering that ${\left\langle L\right\rangle }_{M}(\rho )$ and ${\mathrm{DSF}}_{M}(\rho_{1},\rho_{2})$ are always positive, the minus sign in Eqs. (1) and (3), reflects the fact that an increase in SDM or DSM (i.e., positive values of ΔM(ρ,Δt) or ΔDSM) always corresponds to a decrease in absorption. Finally, we note that both ${\left\langle L\right\rangle }_{M}$ and ${\mathrm{DSF}}_{M}$ were calculated using analytical formulas obtained in the semi-infinite homogeneous medium with extrapolated boundary conditions. For this reason, even these parameters can be considered as “effective” since they differ from the parameters obtained in a two-layered medium. However, for conciseness we will not refer to them as “effective.”

## 3 Results

### 3.1 Measurements *In Vivo*

#### 3.1.1 Effective optical properties of tissue

Effective optical properties of the forearm tissue were measured at baseline (described in Sec. 2.3) before performing either vascular occlusion. These effective optical properties were used to calculate the generalized optical pathlength for intensity (${\left\langle L\right\rangle }_{I}$) and generalized optical pathlength for phase (${\left\langle L\right\rangle }_{\phi }$) at each wavelength (690 and 830 nm) and at each ρ ($\rho_{1}=25\;\mathrm{mm}$ and $\rho_{2}=35\;\mathrm{mm}$) by considering a semi-infinite homogeneous medium (see Table 1). ${\left\langle L\right\rangle }_{I}$ and ${\left\langle L\right\rangle }_{\phi }$ then yield a ${\mathrm{DSF}}_{I}$ and ${\mathrm{DSF}}_{\phi }$ according to Eq. (2). A comparison of the baseline effective optical properties measured on subjects 2 and 4 before the arterial and venous occlusions (subjects 2 and 4 participated in both protocols) shows differences of less than 6%. These differences can be attributed to different hemodynamic conditions and measurement accuracy errors as they exceed the standard deviations over the baseline period reported in Table 1, which represent the measurement precision.

#### 3.1.2 Absorption changes associated with vascular occlusions

**Fig. 2:**
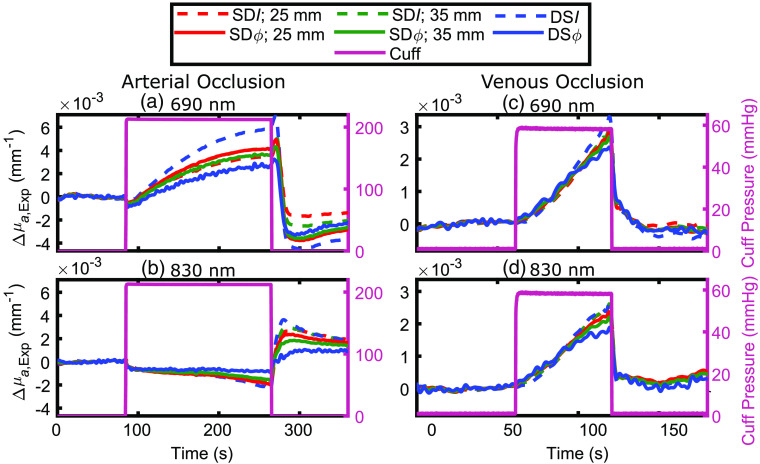
Representative time traces of experimentally measured absorption coefficient change ($\Delta \mu_{a,\mathrm{Exp}}$) during an arterial occlusion [panels (a) and (b): subject 4] and venous occlusion [panels (c) and (d): subject 2]. For each occlusion type, data are shown at wavelengths of 690 nm [panels (a) and (c)] and 830 nm [panels (b) and (d)]. Each subplot contains time traces of $\Delta \mu_{a,\mathrm{Exp}}$ calculated using data from single-distance intensity (SDI) at 25 mm (dashed red) and 35 mm (dashed green), single-distance phase (SDϕ) at 25 mm (solid red) and 35 mm (solid green), dual-slope intensity (DSI) (dashed blue), and dual-slope phase (DSϕ) (solid blue). Co-registered time traces of the measured arm cuff pressure are shown in pink and scaled according to the right axes. All measurements have been low-pass filtered to 0.2 Hz.

Figure 2 shows representative time traces of the experimental $\Delta \mu_{a}$ ($\Delta \mu_{a,\mathrm{Exp}}$) obtained with SD and DS I and ϕ data during an arterial occlusion in subject 4 [Fig. 2(a): 690 nm; Fig. 2(b): 830 nm] and during a venous occlusion in subject 2 [Fig. 2(c): 690 nm; Fig. 2(d): 830 nm]. We remind that $\Delta \mu_{a,\mathrm{Exp}}$ is the effective absorption change, calculated with the methods described in Sec. 2.3. All temporal traces of $\Delta \mu_{a,\mathrm{Exp}}$ have been low-pass filtered to 0.2 Hz to help visualization by removing fast fluctuations and oscillations at the heart rate. Note that greater noise is typically associated with phase versus intensity data. The measured $\Delta \mu_{a,\mathrm{Exp}}$ at the two wavelengths reflect the changes in tissue concentrations of the oxygenated (ΔO) and deoxygenated (ΔD) heme groups during the occlusions.

During an arterial occlusion, the blood flow in and out of the measured limb is blocked, causing an increase in D (ΔD>0) and a decrease of the same magnitude in O (ΔO<0) due to tissue oxygen consumption. At 690 nm, D has a greater extinction coefficient (ϵ) than O and the opposite is true at 830 nm, which translated into an increase in $\mu_{a}$ at 690 nm and a decrease in $\mu_{a}$ at 830 nm, as seen in Figs. 2(a) and 2(b). At the onset of the arterial occlusion, a motion artifact that lasts approximately 1 to 2 s is noticeable at both wavelengths for all data types. This artifact is also visible in DS data types, even though they should be largely insensitive to changes in the optical coupling between sources and detectors.^16^^,^^31^ A possible reason for this artifact to appear in DS data is that the optical coupling change may have occurred faster than the data sampling cycle through all sources and detectors. No motion artifact is visible at the onset of the venous occlusion [Fig. 2(c) and 2(d)], possibly as a result of the lower cuff inflation pressure in this case. During a venous occlusion, only the outflow of blood is blocked (at least initially) from the measured limb so that there is blood pooling and an increase in the total-heme concentration (T) in the tissue. Consequently, $\mu_{a}$ increases at both wavelengths, as shown in Figs. 2(c) and 2(d).

**Fig. 3:**
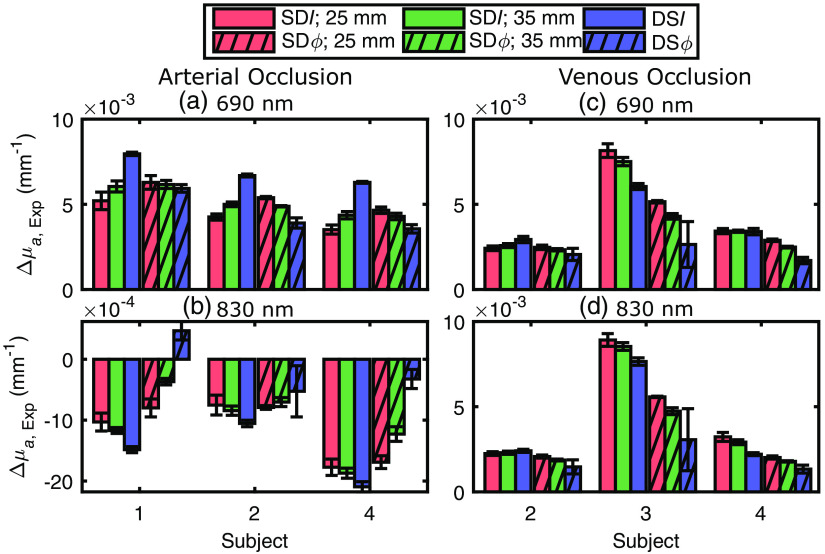
Bar plots depicting the maximum measured absorption coefficient change ($\Delta \mu_{a,\mathrm{Exp}}$) across each subject for an arterial occlusion (a) and (b) and venous occlusion (c) and (d) protocols. Across all panels, measurements using phase (ϕ) have a hatched bar, measurements with intensity (I) are a solid color, measurements with single-distance (SD) at 25 mm are colored in red, measurements with SD at 35 mm are in green, and measurements with dual-slope (DS) are in blue.

Differences in the maximum absorption changes obtained with the six data types (namely SDI and SDϕ at 25 mm, SDI and SDϕ at 35 mm, and DSI and DSϕ) during and after the two vascular occlusion protocols can be seen in Fig. 2. These differences indicate a different spatial sensitivity of the various data types to non-homogeneous hemodynamics in the probed tissue. While Fig. 2 reports data collected on two representative subjects, Fig. 3 summarizes the results for all subjects, protocols, and wavelengths. Figure 3 reports the maximum $\Delta \mu_{a,\mathrm{Exp}}$ resulting from the vascular occlusion, as measured by the difference between the average $\Delta \mu_{a,\mathrm{Exp}}$ over the last 10 s of the occlusion and the average $\Delta \mu_{a}$ at 1 to 2 s after the onset of occlusion (to account for any absorption shifts from baseline due to an artifact occurring at the start of cuff inflation). Error bars in Fig. 3 represent the propagation of standard deviations of measurements over time (1 to 2 s after the onset of occlusion and last 10 s of occlusion).

While the experimental data have been analyzed under the simplifying assumption that the investigated tissue is spatially homogeneous (with baseline effective optical properties reported in Table 1 and effective absorption changes), the layered anatomical structure of the skin, adipose tissue, and skeletal muscle accounts for in-homogeneous baseline optical properties as well as in-homogeneous hemodynamic responses to vascular occlusions. One may think that, regardless of the baseline optical properties, collecting SD optical data at longer source–detector distances would always increase the sensitivity to hemodynamic changes in deeper tissue layers. In the case of homogeneous semi-infinite media with layered absorption changes, this is usually the case, and we have also shown that the sensitivity to absorption changes in the bottom layer is greater for DSϕ than DSI data.^16^

For all three subjects in the arterial occlusion protocol [Figs. 3(a) and 3(b)] and for subject 2 in the venous occlusion protocol [Figs. 3(c) and 3(d)], the maximum absorption changes measured with I data seem to indicate a greater absorption change (in absolute value) deeper in the tissue. As described in the introduction, this behavior is consistent with a higher increase of oxygen consumption in muscles than in adipose tissue. In fact, the measured absorption change progressively increases in absolute value when obtained from SDI at 25 mm, SDI at 35 mm, and DSI. However, the opposite is true for ϕ data, in which case the measured absorption change progressively decreases in absolute value when obtained from SDϕ at 25 mm, SDϕ at 35 mm, and DSϕ. This paradoxical result is assigned to non-homogeneous baseline optical properties in combination with in-homogeneous hemodynamic changes in the investigated tissue. We also note the positive change (increase) in the absorption coefficient at 830 nm in subject 1 measured with DSϕ during an arterial occlusion [Fig. 3(b)], which is assigned to a spatially heterogeneous increase in heme concentration (resulting from blood redistribution) to which DSϕ data is most sensitive.

No paradoxical behavior is observed in subjects 3 and 4 in the venous occlusion protocol [Figs. 3(c) and 3(d)]. The measured values of $\Delta \mu_{a,\mathrm{Exp}}$ with the different data types are consistent with a smaller increase in blood volume in deeper tissue (muscle) versus superficial tissue (adipose tissue). This is consistent with reported higher blood flow in adipose tissue than in muscle. In fact, the measured absorption change progressively decreases when it is obtained from SDI at 25 mm, SDI at 35 mm, DSI, SDϕ at 25 mm, SDϕ at 35 mm, and DSϕ. To investigate whether in-homogeneous optical properties at baseline and in-homogeneous hemodynamics during vascular occlusion in the superficial adipose tissue and in the deeper skeletal muscle tissue can account for the observed results, we have modeled the tissue as a two-layer medium as presented in Sec. 3.2.

#### 3.1.3 Oxygen consumption and blood flow obtained from initial absorption change rates during an arterial or venous occlusion, respectively

Absorption changes at 690 and 830 nm were translated into tissue ΔO and ΔD using the molar extinction coefficient of oxy- and deoxyhemoglobin (each containing four heme groups). Tissue oxygen consumption was obtained from the initial temporal slope of (ΔD−ΔO)/2 after the start of arterial occlusion. For all subjects, the calculated oxygen consumption was in the range of 0.95 to 2.2  μmol/(100  mL)/min. Tissue blood flow as obtained from the initial temporal slope of ΔT after the start of the venous occlusion. The range for calculated blood flow was 0.48 to 4.7  mL/(100  mL)/min.

### 3.2 Two-layer Simulation

The simulations based on diffusion theory for a two-layered medium intend to investigate the effect of baseline optical properties and absorption changes in the two layers on the effective absorption changes obtained with different data types.

#### 3.2.1 Effect of baseline scattering in the two layers

**Fig. 4:**
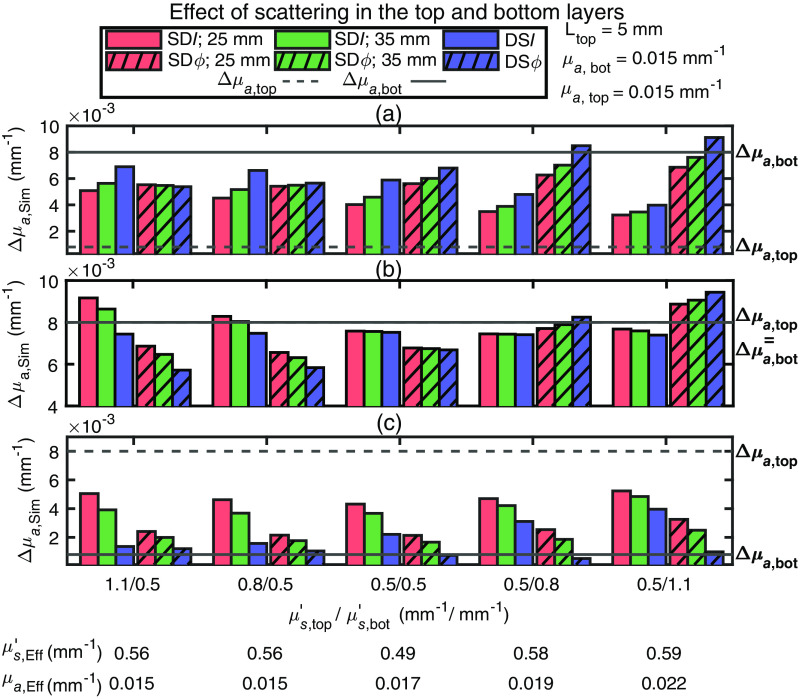
Simulated absorption coefficient change ($\Delta \mu_{a,\mathrm{Sim}}$) obtained with different data types collected on two-layered medium with a different baseline reduced scattering coefficient in the top layer ($\mu_{s,\text{top}}^{\prime }$) and bottom layer ($\mu_{s,\mathrm{bot}}^{\prime }$). For all simulations, the top layer thickness ($L_{\text{top}}$) was 5 mm, and the absorption coefficient for the top layer ($\mu_{a,\mathrm{top}}$) and bottom layer ($\mu_{a,\mathrm{bot}}$) were both set to $0.015\;{\mathrm{mm}}^{-1}$. In all panels, measurements using phase (ϕ) data are represented by a hatched bar, measurements using intensity (I) data by a solid bar, measurements using single-distance (SD) at 25 mm are colored in red, measurements using SD at 35 mm are in green, and measurements using dual-slope (DS) are in blue. The absorption coefficient change for the top ($\Delta \mu_{a,\text{top}}$) and bottom ($\Delta \mu_{a,\mathrm{bot}}$) layers were simulated for the cases of (a) $\Delta \mu_{a,\text{top}}<\Delta \mu_{a,\mathrm{bot}}\;(\Delta \mu_{a,\text{top}}=0.0008\;{\mathrm{mm}}^{-1},\;\Delta \mu_{a,\mathrm{bot}}=0.008\;{\mathrm{mm}}^{-1})$; (b) $\Delta \mu_{a,\text{top}}=\Delta \mu_{a,\mathrm{bot}}\;(\Delta \mu_{a,\text{top}}=\Delta \mu_{a,\mathrm{bot}}=0.008\;{\mathrm{mm}}^{-1})$; and (c) $\Delta \mu_{a,\text{top}}>\Delta \mu_{a,\mathrm{bot}}\;(\Delta \mu_{a,\text{top}}=0.008\;{\mathrm{mm}}^{-1},\Delta \mu_{a,\mathrm{bot}}=0.0008\;{\mathrm{mm}}^{-1})$. These values of baseline optical properties and absorption changes were chosen to be consistent with the results of *in vivo* measurements. The bottom of the horizontal axis label reports the effective homogeneous absorption coefficient ($\mu_{a,\mathrm{Eff}}$) and reduced scattering coefficient ($\mu_{s,\mathrm{Eff}}^{\prime }$) obtained from the combination of DSI and DSϕ data.

Simulated $\Delta \mu_{a}$ ($\Delta \mu_{a,\mathrm{Sim}}$) from data generated in a two-layered medium for different values of the top and bottom layer $\mu_{s}^{\prime }$ ($\mu_{s,\text{top}}^{\prime }$ and $\mu_{s,\mathrm{bot}}^{\prime }$, respectively) are shown in Fig. 4. Specifically, we considered cases of greater $\mu_{s}^{\prime }$ in the top layer, the same $\mu_{s}^{\prime }$ in the two layers, and smaller $\mu_{s}^{\prime }$ in the top layer, according to the x-axis of Fig. 4. A homogeneous $\mu_{a}$ was considered so that the top ($\mu_{a,\text{top}}$) and bottom ($\mu_{a,\mathrm{bot}}$) absorption coefficients were both set to $0.015\;{\mathrm{mm}}^{-1}$, and the top layer thickness ($L_{\text{top}}$) was set to 5 mm. Furthermore, we examined three cases: $\Delta \mu_{a}$ smaller in the top layer than in the bottom layer [Fig. 4(a)], the same $\Delta \mu_{a}$ in the two layers [Fig. 4(b)], and $\Delta \mu_{a}$ greater in the top layer than in the bottom layer [Fig. 4(c)].

When $\Delta \mu_{a,\text{top}}<\Delta \mu_{a,\mathrm{bot}}$ [Fig. 4(a)], when $\mu_{s,\text{top}}^{\prime }/\mu_{s,\mathrm{bot}}^{\prime }$ decreases from values > 1 to values less than 1 (i.e., when the top layer transitions from being more scattering to being less scattering than the bottom layer), the effective $\Delta \mu_{a,\mathrm{Sim}}$ obtained with ϕ data increases, whereas the effective $\Delta \mu_{a,\mathrm{Sim}}$ obtained with I data decreases. This shows that as the scattering of the top layer decreases, the phase data becomes more representative of the absorption changes in the bottom layer, and intensity data become more representative of absorption changes in the top layer. A physical interpretation of this result is that as the scattering of the bottom-layer increases in relation to the scattering of the top layer, the spatial sensitivity of intensity data shifts toward the top layer, whereas the sensitivity for phase data shifts toward the bottom layer. This is an example of the complementary information provided by intensity data in relation to the two layers of a layered medium, which is the basis for this work about leveraging FD-NIRS data for discrimination of adipose tissue and skeletal muscle hemodynamics.

When $\Delta \mu_{a,\text{top}}=\Delta \mu_{a,\mathrm{bot}}$ [Fig. 4(b)], if $\mu_{s,\text{top}}^{\prime }>\mu_{s,\mathrm{bot}}^{\prime }$ SDI data overestimate the true $\Delta \mu_{a}$ while all other data types underestimate the true $\Delta \mu_{a}$. As the scattering mismatch between the two layers decreases, $\Delta \mu_{a,\mathrm{Sim}}$ obtained with SDI approaches the true $\Delta \mu_{a}$ and, in the homogeneous case all $\Delta \mu_{a,\mathrm{Sim}}$ are less than the true $\Delta \mu_{a}$ (as a result of a relatively large $\Delta \mu_{a}$ and the nonlinear dependence of the various data types changes on $\Delta \mu_{a}$). As $\mu_{s,\mathrm{top}}^{\prime }$ becomes smaller than $\mu_{s,\mathrm{bot}}^{\prime }$, $\Delta \mu_{a,\mathrm{Sim}}$ obtained with ϕ data becomes greater than $\Delta \mu_{a,\mathrm{Sim}}$ obtained with I data. At the smallest value of $\mu_{s,\mathrm{top}}^{\prime }/\mu_{s,\mathrm{bot}}^{\prime }$, it is $\Delta \mu_{a,\mathrm{Sim}}$ obtained with phase data that overestimates the true $\Delta \mu_{a}$. Lastly, when $\Delta \mu_{a,\text{top}}>\Delta \mu_{a,\mathrm{bot}}$ [Fig. 4(c)], varying the scattering mismatch between the two layers has no major impact, with the most notable effects being a consistently good agreement between $\Delta \mu_{a,\mathrm{Sim}}$ obtained with DSϕ data and $\Delta \mu_{a,\mathrm{bot}}$, and a worsening agreement between $\Delta \mu_{a,\mathrm{bot}}$ and $\Delta \mu_{a,\mathrm{Sim}}$ obtained with DSI data as the ratio $\mu_{s,\text{top}}^{\prime }/\mu_{s,\mathrm{bot}}^{\prime }$ decreases.

Of the cases considered in Fig. 4, there is only one that reproduces the paradoxical behavior observed *in vivo* during arterial occlusion (in all three subjects) and venous occlusion (in subject 2): it is the case of $\mu_{s,\text{top}}^{\prime }>\mu_{s,\mathrm{bot}}^{\prime }$ and $\Delta \mu_{a,\text{top}}<\Delta \mu_{a,\mathrm{bot}}$ [Fig. 4(a)]. For the venous occlusion protocol in subjects 3 and 4, two possible scenarios from the two-layer simulations reproduced the *in vivo* results. They are (1) $\mu_{s,\text{top}}^{\prime }>\mu_{s,\mathrm{bot}}^{\prime }$ and $\Delta \mu_{a,\text{top}}\geq \Delta \mu_{a,\mathrm{bot}}$ and (2) $\mu_{s,\text{top}}^{\prime }<\mu_{s,\mathrm{bot}}^{\prime }$ and $\Delta \mu_{a,\text{top}}>\Delta \mu_{a,\mathrm{bot}}$.

As a final comment on the results of Fig. 4, we note that in the case of a homogeneous medium and a homogeneous absorption change [the set of bars in the center of Fig. 4(b)], the underestimation of the true $\Delta \mu_{a}$ by $\Delta \mu_{a,\mathrm{Sim}}$ is due to the non-linear dependence on $\Delta \mu_{a}$ of $\Delta \ln(\rho^{2}I)$, Δϕ, and associated slope changes. To mimic the experimental results, in Fig. 4(b) we have used a relatively large $\Delta \mu_{a}$ (about 50% of the background $\mu_{a}$), which is beyond the linear approximation intrinsic in the DPF and differential slope factor (DSF) approach. We have verified that using a much smaller $\Delta \mu_{a}$ (<0.1% of the background $\mu_{a}$), $\Delta \mu_{a,\mathrm{Sim}}$ is about equal to the true $\Delta \mu_{a}$ for all data types.

#### 3.2.2 Effect of baseline absorption in the two layers

**Fig. 5:**
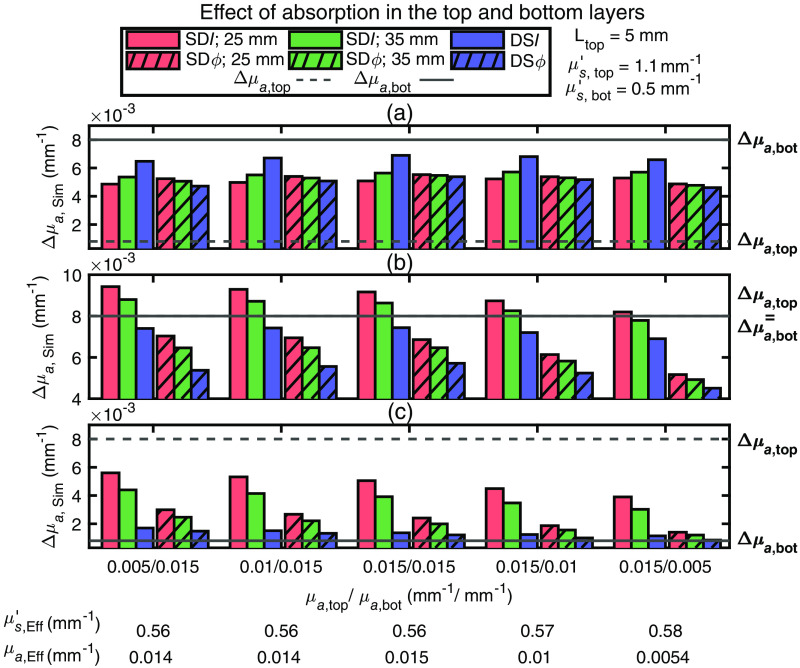
Simulated absorption coefficient change ($\Delta \mu_{a,\mathrm{Sim}}$) obtained with different data types collected on two-layered medium with different baseline absorption coefficient in the top layer ($\mu_{a,\text{top}}$) and bottom layer ($\mu_{a,\text{bot}}$). For all simulations, the top layer thickness ($L_{\text{top}}$) was 5 mm, and the reduced scattering coefficient ($\mu_{s}^{\prime }$) was set to $1.1\;{\mathrm{mm}}^{-1}$ for the top layer ($\mu_{s,\text{top}}^{\prime }$) and to $0.5\;{\mathrm{mm}}^{-1}$ for the bottom layer ($\mu_{s,\mathrm{bot}}^{\prime }$). In all panels, measurements using phase (ϕ) data are represented by a hatched bar, measurements using intensity (I) data by a solid bar, measurements using single-distance (SD) at 25 mm are colored in red, measurements using SD at 35 mm are in green, and measurements using dual-slope (DS) are in blue. The absorption coefficient change for the top ($\Delta \mu_{a,\text{top}}$) and bottom ($\Delta \mu_{a,\mathrm{bot}}$) layers were simulated for the cases of (a) $\Delta \mu_{a,\mathrm{top}}<\Delta \mu_{a,\mathrm{bot}}\;(\Delta \mu_{a,\text{top}}=0.0008\;{\mathrm{mm}}^{-1},\;\Delta \mu_{a,\mathrm{bot}}=0.008\;{\mathrm{mm}}^{-1})$; (b) $\Delta \mu_{a,\text{top}}=\Delta \mu_{a,\mathrm{bot}}\;(\Delta \mu_{a,\text{top}}=\Delta \mu_{a,\mathrm{bot}}=0.008\;{\mathrm{mm}}^{-1})$; and (c) $\Delta \mu_{a,\text{top}}>\Delta \mu_{a,\mathrm{bot}}\;(\Delta \mu_{a,\text{top}}=0.008\;{\mathrm{mm}}^{-1},\Delta \mu_{a,\mathrm{bot}}=0.0008\;{\mathrm{mm}}^{-1})$. These values of baseline optical properties and absorption changes were chosen to be consistent with the results of *in vivo* measurements. The bottom of the horizontal axis label reports the effective homogeneous absorption coefficient ($\mu_{a,\mathrm{Eff}}$) and reduced scattering coefficient ($\mu_{s,\mathrm{Eff}}^{\prime }$) obtained from the combination of DSI and DSϕ data.

Simulated absorption changes ($\Delta \mu_{a,\mathrm{Sim}}$) from data generated in a two-layered medium for different values of the top and bottom layer $\mu_{a}$ are shown in Fig. 5. In this simulation, $\mu_{s,\text{top}}^{\prime }=1.1\;{\mathrm{mm}}^{-1}$, $\mu_{s,\mathrm{bot}}^{\prime }=0.5\;{\mathrm{mm}}^{-1}$, and $L_{\text{top}}=5\;\mathrm{mm}$. These values of $\mu_{s}^{\prime }$ in the two layers were chosen as they were most representative of the *in vivo* results observed in both occlusions protocols, as discussed in relation to Fig. 4. From Fig. 5, it is seen that the values of $\mu_{a,\text{top}}$ and $\mu_{a,\mathrm{bot}}$ do not significantly impact the qualitative relationship between the absorption changes obtained with the six data types. In the case $\Delta \mu_{a,\text{top}}>\Delta \mu_{a,\mathrm{bot}}$ [Fig. 5(c)], it is worth noting that the absorption change obtained with all the data types tends to approach the true $\Delta \mu_{a,\mathrm{bot}}$ as $\mu_{a,\text{top}}/\mu_{a,\mathrm{bot}}$ increases.

#### 3.2.3 Effect of top layer thickness

**Fig. 6:**
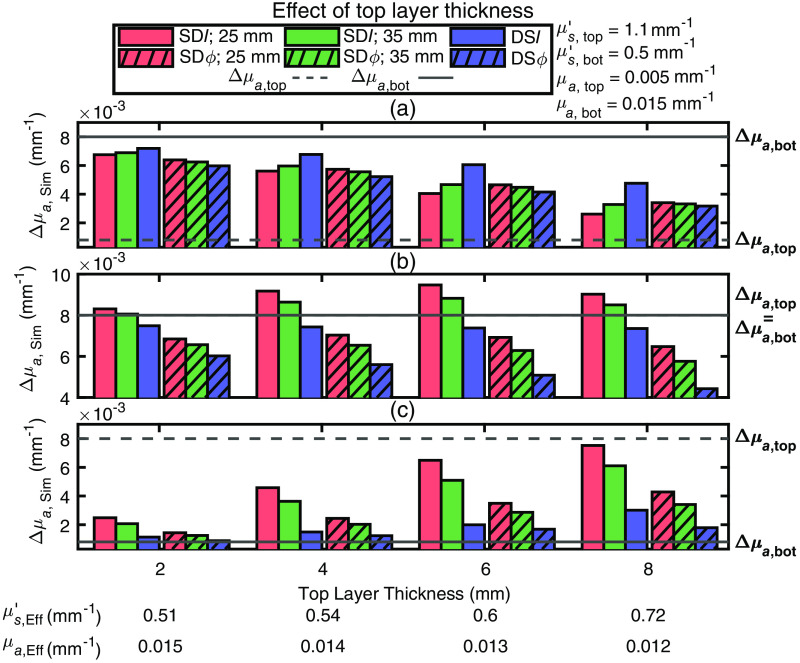
Simulated absorption coefficient change ($\Delta \mu_{a,\mathrm{Sim}}$) obtained with different data types collected on a two-layered medium with different values of the top-layer thickness ($L_{\text{top}}$). For all simulations, the absorption coefficient of the top layer ($\mu_{a,\text{top}}$) was set to $0.005\;{\mathrm{mm}}^{-1}$ and the one of the bottom layer ($\mu_{a,\mathrm{bot}}$) was set to $0.015\;{\mathrm{mm}}^{-1}$, whereas the reduced scattering coefficient was set to $1.1\;{\mathrm{mm}}^{-1}$ for the top layer ($\mu_{s,\text{top}}^{\prime }$) and to $0.5\;{\mathrm{mm}}^{-1}$ for the bottom layer ($\mu_{s,\mathrm{bot}}^{\prime }$). In all panels, measurements using phase (ϕ) data are represented by a hatched bar, measurements using intensity (I) data by a solid bar, measurements using single-distance (SD) at 25 mm are colored in red, measurements using SD at 35 mm are in green, and measurements using dual-slope (DS) are in blue. The absorption coefficient change for the top ($\Delta \mu_{a,\mathrm{top}}$) and bottom ($\Delta \mu_{a,\mathrm{bot}}$) layers were simulated for the cases of (a) $\Delta \mu_{a,\text{top}}<\Delta \mu_{a,\mathrm{bot}}\;(\Delta \mu_{a,\text{top}}=0.0008\;{\mathrm{mm}}^{-1},\;\Delta \mu_{a,\mathrm{bot}}=0.008\;{\mathrm{mm}}^{-1})$; (b) $\Delta \mu_{a,\text{top}}=\Delta \mu_{a,\mathrm{bot}}\;(\Delta \mu_{a,\text{top}}=\Delta \mu_{a,\mathrm{bot}}=0.008\;{\mathrm{mm}}^{-1})$; and (c) $\Delta \mu_{a,\text{top}}<\Delta \mu_{a,\mathrm{bot}}\;(\Delta \mu_{a,\text{top}}=0.008\;{\mathrm{mm}}^{-1},\;\Delta \mu_{a,\mathrm{bot}}=0.0008\;{\mathrm{mm}}^{-1})$. These values of baseline optical properties and absorption changes were chosen to be consistent with the results of *in vivo* measurements. The bottom of the horizontal axis label reports the effective homogeneous absorption coefficient ($\mu_{a,\mathrm{Eff}}$) and reduced scattering coefficient ($\mu_{s,\mathrm{Eff}}^{\prime }$) obtained from the combination of DSI and DSϕ data.

Simulated absorption changes ($\Delta \mu_{a,\mathrm{Sim}}$) from data generated in a two-layered medium for different values of the top layer thickness (in the range 2 to 8 mm) are shown in Fig. 6. In the cases where $\Delta \mu_{a,\text{top}}<\Delta \mu_{a,\mathrm{bot}}$ [Fig. 6(a)] or $\Delta \mu_{a,\text{top}}>\Delta \mu_{a,\mathrm{bot}}$ [Fig. 6(c)], as the top layer thickness increases $\Delta \mu_{a,\mathrm{Sim}}$ tends toward $\Delta \mu_{a,\text{top}}$ for every data type. This is an intuitive result since a thicker superficial layer is expected to result in a greater sensitivity to it. The case where $\Delta \mu_{a,\text{top}}=\Delta \mu_{a,\mathrm{bot}}$ [Fig. 6(b)] yields less intuitive results in which $\Delta \mu_{a,\mathrm{Sim}}$ obtained with I data show a closer agreement to the true homogeneous $\Delta \mu_{a}$ than $\Delta \mu_{a,\mathrm{Sim}}$ obtained with ϕ data. This latter result was also observed in the case of $\mu_{s,\text{top}}^{\prime }/\mu_{s,\mathrm{bot}}^{\prime }\leq 1$, regardless of the baseline absorption coefficients of the two layers [see Figs. 4(b) and 5(b)].

## 4 Discussion

### 4.1 Baseline *In Vivo* Optical Properties

The effective homogeneous baseline optical properties, measured on the human forearm by a combination of DSI and DSϕ data analyzed with diffusion theory for a semi-infinite homogeneous medium, are reported in Table 1 for all subjects and wavelengths (690 and 830 nm). The $\mu_{a}$ and $\mu_{s}^{\prime }$ values are in the range of 0.015 to $0.019\;{\mathrm{mm}}^{-1}$ and 0.48 to $0.60\;{\mathrm{mm}}^{-1}$, respectively, across the various subjects. These values agree with optical properties measured non-invasively on human skeletal muscle (specifically, the vastus lateralis) using FD-NIRS.^9^ With an assumed 75% water content, these baseline absorption coefficients at the two wavelengths result in the following range of values for the heme-related quantities: O: 40 to 56  μm; D: 24 to 35  μm; T: 69 to 82  μm; and tissue saturation (${\mathrm{StO}}_{2}=O/T$): 55% to 70%. These values are also comparable to previous FD-NIRS measurements^10^^,^^13^^,^^32^ even though ${\mathrm{StO}}_{2}$ values are at the lower end of the range of values typically reported, possibly due to correction for water absorption.^33^ In this work, the relevance of baseline effective optical properties is for the determination of ${\left\langle L\right\rangle }_{I}$ and ${\left\langle L\right\rangle }_{\phi }$ used to calculate the $\Delta \mu_{a}$ from SD and DS data according to Eqs. (1) and (3).

### 4.2 Absorption Changes and Associated Hemodynamics *In Vivo*

The measured absorption changes of the forearm tissue in response to vascular occlusions vary for different data types (Fig. 3). Because these absorption changes reflect blood accumulation (during a venous occlusion) and blood deoxygenation (during an arterial occlusion), they are the basis for measurements of muscle blood flow (using a venous occlusion protocol) and muscle oxygen consumption (using an arterial occlusion protocol). Even though the experimental $\Delta \mu_{a}$ values reported in Fig. 3 are maximum changes during vascular occlusions, whereas blood flow and oxygen consumption are obtained from initial rates of change of ΔO and ΔD during vascular occlusion, we translated the initial rates of change in our results to oxygen consumption and blood flow. The range of values observed for rest blood flow was 0.48 to 4.7  mL/(100  mL)/min and for rest oxygen consumption was 0.95 to 2.2  μmol/(100  mL)/min or 0.02 to 0.05  mL/(100  mL)/min. These values are comparable to those previously reported using NIRS measurements during venous or arterial occlusion, respectively.^6^^,^^32^^,^^34^^,^^35^ The emphasis of this work is on taking advantage of the different values of $\Delta \mu_{a}$ obtained with the different data types considered here (SDI, SDϕ, DSI, and DSϕ) to be able to discriminate the superficial (skin + adipose tissue) and deeper (skeletal muscle) hemodynamics. The goal of this research is to perform hemodynamic measurements that are representative of skeletal muscle considering the fact that adipose tissue and muscle tissue have different baseline optical properties and feature different hemodynamics during external perturbations or physiological processes.

### 4.3 Theoretical Simulations in a Two-Layered Medium

We used diffusion theory to simulate data in a two-layered medium of known geometrical and optical properties to reproduce key features of experimental data collected on the forearm muscle during vascular occlusion protocols. To make a fair comparison, the same methods for calculating $\Delta \mu_{a}$ was used for simulated and experimental data. A fundamental approach in these methods was that the values of ⟨L⟩, which are used to find the DPF and DSF for I and ϕ data, were obtained using effective homogeneous optical properties. Previous work has studied the dependence of the SDI DPF (obtained from the mean photon time-of-flight measured with TD-NIRS) on the optical properties and top layer thickness of a two-layered medium^36^ but did not study the effect of assuming a homogeneous medium in the calculation of the DPF. To see the impact on our results of obtaining DPF and DSF under the assumption that the tissue is homogeneous, we re-ran the simulations reported in Fig. 4 using the correct DPF and DSF (i.e., using ${\left\langle L\right\rangle }_{M}$ values obtained from the actual optical properties of a two-layered medium). Our most surprising and counter-intuitive result is the one reported in Fig. 4(a), where the $\Delta \mu_{a}$ (obtained with intensity and phase data) exhibit an opposite dependence on the source–detector distance when $\mu_{s,\text{top}}^{\prime }>\mu_{s,\mathrm{bot}}^{\prime }$. Our goal was to explore whether this result may be due to the incorrect assumption of a homogeneous medium in the determination of DPF and DSF.

**Fig. 7:**
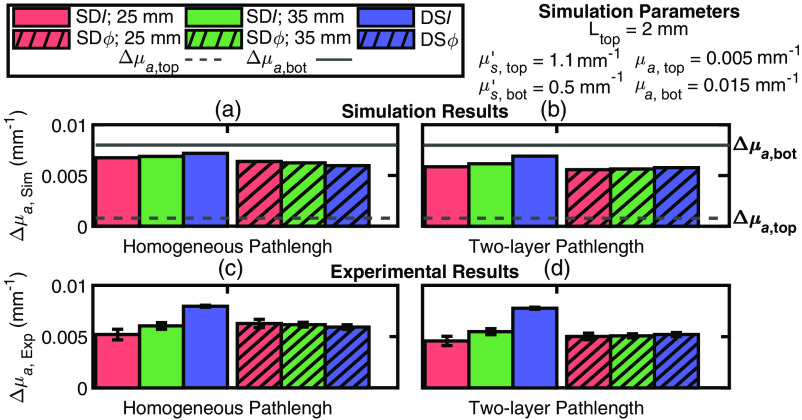
Comparison of the absorption coefficient change ($\Delta \mu_{a}$) obtained from a generalized mean optical pathlength (⟨L⟩) assuming either a homogeneous medium [panels (a) and (c)] or a two-layered medium [panels (b) and (d)] in simulations ($\Delta \mu_{a,\mathrm{Sim}}$) [panels (a) and (b)] and in experiments *in vivo* ($\Delta \mu_{a,\mathrm{Exp}}$) [panels (c) and (d)] on subject 1 at 690 nm during an arterial occlusion. For all simulations, the top layer thickness ($L_{\text{top}}$) was 2 mm, the absorption coefficient ($\mu_{a}$) in the top layer ($\mu_{a,\text{top}}$) was $0.005\;{\mathrm{mm}}^{-1}$ and in the bottom layer ($\mu_{a,\mathrm{bot}}$) was $0.015\;{\mathrm{mm}}^{-1}$, and the reduced scattering coefficient ($\mu_{s}^{\prime }$) for the top layer ($\mu_{s,\text{top}}^{\prime }$) was $1.1\;{\mathrm{mm}}^{-1}$ and in the bottom layer ($\mu_{s,\mathrm{bot}}^{\prime }$) was $0.5\;{\mathrm{mm}}^{-1}$.

For the specific case of Fig. 4(a) with $\mu_{s,\text{top}}^{\prime }=1.1\;{\mathrm{mm}}^{-1}$ and $\mu_{s,\mathrm{bot}}^{\prime }=0.5\;{\mathrm{mm}}^{-1}$, Fig. 7(a) shows simulations using DPF and DSF calculated from the effective homogeneous optical properties [as in Fig. 4(a)], whereas Fig. 7(b) shows simulations using DPF and DSF calculated using the correct two-layer medium optical properties. Using the correct two-layer absorption changes [Fig. 7(b)], the values of $\Delta \mu_{a}$ obtained with the different data types show the consistent behavior of increasing source–detector distance (and being maximal in the DS configuration) for both I and ϕ data, whereas using the homogeneous pathlength [Fig. 7(a)] results in an opposite behavior of intensity and phase data. This result is now fully consistent with a greater absorption change in the bottom layer and shows that the paradoxical result of Fig. 4(a) for $\mu_{s,\text{top}}^{\prime }=1.1\;{\mathrm{mm}}^{-1}$ and $\mu_{s,\mathrm{bot}}^{\prime }=0.5\;{\mathrm{mm}}^{-1}$ may indeed follow from the use of effective homogeneous optical properties instead of the correct two-layer properties in the calculation of DPF and DSF. To further test this result on experimental data, we calculated $\Delta \mu_{a,\mathrm{Exp}}$ for one subject (subject 1) and one wavelength (690 nm) during an arterial occlusion using the DPF and DSF calculated for the two-layer optical properties that best matched the experimental data [as also used in Figs. 7(a) and 7(b)]. The ⟨L⟩ values derived for I (${\left\langle L\right\rangle }_{I}$) and ϕ (${\left\langle L\right\rangle }_{\phi }$) assuming homogeneous and two-layered optical properties are reported in Table 2. Using the DPF and DSF for a homogeneous medium results in values of $\Delta \mu_{a,\mathrm{Exp}}$ [reported in Fig. 7(c)] that replicate those of Fig. 4(a). Using the DPF and DSF for a two-layered medium modifies the values of $\Delta \mu_{a,\mathrm{Exp}}$ [as reported in Fig. 7(d)] similarly to the simulation in Fig. 7(b). This is one particular case in which changing the method of calculating ⟨L⟩ resulted in a significant difference in our results, but this was not the case for all other subjects.

**Table 2:** Generalized mean optical pathlength for intensity (${\left\langle L\right\rangle }_{I}$) and phase (${\left\langle L\right\rangle }_{\phi }$) at 690 nm using either a homogeneous medium having optical properties calculated from experimental data, or a two-layered medium. For the two-layer medium, the top layer thickness ($L_{\text{top}}$) was 2 mm, the absorption coefficient in the top layer ($\mu_{a,\text{top}}$) was $0.005\;{\mathrm{mm}}^{-1}$ and in the bottom layer ($\mu_{a,\mathrm{bot}}$) was $0.015\;{\mathrm{mm}}^{-1}$, the reduced scattering coefficient for the top layer ($\mu_{s,\text{top}}^{\prime }$) was $1.1\;{\mathrm{mm}}^{-1}$, and in the bottom layer ($\mu_{s,\mathrm{bot}}^{\prime }$) was $0.5\;{\mathrm{mm}}^{-1}$. Here, the source–detector distances (ρ) are 25 mm $\left(\rho_{1}\right)$ and 35 mm ($\rho_{2}$).

Sub 1 at 690 nm	${\left\langle L\right\rangle }_{I}$ (mm)		${\left\langle L\right\rangle }_{\phi }$ (mm)	
	$\rho_{1}$	$\rho_{2}$	$\rho_{1}$	$\rho_{2}$
Homogeneous medium	106	153	10.3	15.6
Two-layer medium	121	168	12.9	19.8

In the absence of a knowledge of the optical properties of the top and bottom layer, the assumption of a homogeneous medium to calculate the DPF and DSF is required. Several studies, particularly in CW-NIRS, have assumed a wavelength-independent DPF^6^^,^^7^^,^^12^ taken from the literature, which will introduce errors in the measured values of $\Delta \mu_{a}$ and in their wavelength dependence.

### 4.4 Limitations of Modeling Based on a Two-Layered Medium

Modeling tissue as a two-layered medium assumes lateral homogeneity. Lateral in-homogeneities in tissue optical properties and in tissue hemodynamics under the optical probe can arise for several reasons, including physiological/anatomical differences or uneven contact pressure between the probe and tissue. Spatial mapping of skeletal muscle has shown variability across muscle regions.^9^^,^^34^^,^^37^ The datasets for this study resulted in similar $\Delta \mu_{a,\mathrm{Exp}}$ for the two SDs at the same distance but on opposite sides on the probe [Fig. 1(c)], which supports the assumption of lateral homogeneity. However, in the presence of lateral heterogeneity in the tissue optical properties, tissue hemodynamics, and/or probe-tissue contact pressure, the assumption of lateral homogeneity intrinsic in the two-layered model may lead to incorrect results. Special care must be taken in applying the optical probe with even pressure on the muscle tissue, and the lateral homogeneity of the measured hemodynamics should be verified before applying the methods presented here.

### 4.5 Implications of this Study for Optical Measurements of Muscle Hemodynamics

Comparison between the experimental results *in vivo* for the various data types (Fig. 3) and the simulations on a two-layered medium (Figs. 4–6) lead to the following conclusions for the baseline optical properties and the absorption changes in the top and bottom tissue layers.

1. In the case of an arterial occlusion, $\mu_{s,\text{top}}^{\prime }>\mu_{s,\mathrm{bot}}^{\prime }$ and $\left|\Delta \mu_{a,\text{top}}|<|\Delta \mu_{a,\mathrm{bot}}\right|$ (see Fig. 4), and $\mu_{a,\text{top}}\leq \mu_{a,\mathrm{bot}}$ [see Fig. 5(a)]. These results for baseline optical properties are consistent with the higher scattering and lower absorption previously reported for adipose tissue versus muscle tissue.^8^^,^^38^^,^^39^ The lower absorption change in the top (adipose) layer versus the bottom (muscle) layer during arterial occlusion implies a lower oxygen consumption in adipose versus muscle tissue at rest, which has previously been reported.^6^^,^^8^^,^^24^^,^^40^
2. In the case of venous occlusion, the *in vivo* results in subject 2 are in line with the arterial occlusion results above, whereas in subjects 3 and 4 they are consistent with either of these two scenarios: (1) $\mu_{s,\text{top}}^{\prime }>\mu_{s,\mathrm{bot}}^{\prime }$ and $\Delta \mu_{a,\text{top}}\geq \Delta \mu_{a,\mathrm{bot}}$ [see Figs. 4(b) and 4(c)] or (2) $\mu_{s,\text{top}}^{\prime }<\mu_{s,\mathrm{bot}}^{\prime }$ and $\Delta \mu_{a,\mathrm{top}}\;>\;\Delta \mu_{a,\mathrm{bot}}$ [Fig. 4(c)]. For consistency with the baseline optical properties observed in the arterial occlusion case, scenario (1) is the selected one. The greater or comparable absorption change in the top (adipose) layer versus the bottom (muscle) layer during venous occlusion implies a greater or comparable blood flow in adipose versus muscle tissue at rest. This result is in line with a previous study that reported a constant (or slightly increasing) measured blood flow with NIRS as a function of adipose layer thickness.^6^
3. The top layer thickness in the range 2 to 8 mm (Fig. 6) significantly impacted the magnitude of $\Delta \mu_{a}$ obtained using different data types, but only marginally impacted their relative values.

The results presented here demonstrate the different impact of baseline optical properties and absorption changes of a two-layered medium on the various data types measured with FD-NIRS in a DS configuration. Therefore, these results suggest the feasibility of discriminating baseline optical properties and hemoglobin concentration changes in adipose and muscle tissue by fitting measured and simulated $\Delta \mu_{a}$ obtained with the various data types. The results presented here do not identify a single data type (SDI, SDϕ, DSI, and DSϕ) that is most effective at sensing the deeper absorption changes (in muscle tissue) with minimal sensitivity to superficial absorption changes (in skin and adipose tissue). While previous work identified DSϕ as a data type that should indeed feature an optimal preferential sensitivity to deeper tissue,^19^ such previous work assumed optical properties that are homogeneous at baseline. In this work, we have shown that layered optical properties, and especially a top layer that is more scattering than the bottom layer, may significantly impact the spatial regions of sensitivity of the various data types considered here depending on the top layer thickness and the specific distribution of optical properties at baseline. Consequently, rather than identifying a single most effective data type for non-invasive muscle measurements, we propose to take advantage of the collective information content of all data types collected by DS FD-NIRS, specifically $\mathrm{SDI}(\rho_{1})$, $\mathrm{SD}I(\rho_{2})$, $\mathrm{SD}\phi (\rho_{1})$, $\mathrm{SD}\phi (\rho_{2})$, DSI, and DSϕ, to discriminate the superficial and deeper tissue hemodynamics. This may be done by fitting the data with a two-layered model of the investigated tissue to obtain baseline and dynamic optical properties of the two layers (with the top layer thickness measured independently or also treated as a fitting parameter). While this method is rigorous, it may require data at more than two source–detector distances for an accurate determination of all fitting parameters. Alternatively, we could use the two-layer model in a different way. First, the DS FD-NIRS data are used to measure effective baseline optical properties ($\mu_{a,\mathrm{Eff}}$ and $\mu_{s,\mathrm{Eff}}^{\prime }$ from which DPF and DSF values are obtained), the top layer thickness is measured independently (for example by a skinfold caliper or ultrasound imaging), and the effective $\Delta \mu_{a}$ values obtained with the different data types are then measured during protocols that elicit tissue hemodynamic and/or oxygenation changes. The set of effective optical properties and effective $\Delta \mu_{a}$ values are then fit with those obtained/assumed from two-layered media, using the four fitting parameters of $\mu_{s,\text{top}}^{\prime }$, $\mu_{s,\mathrm{bot}}^{\prime }$, $\Delta \mu_{a,\text{top}}$, and $\Delta \mu_{a,\mathrm{bot}}$ (scattering changes may be neglected, i.e., $\Delta \mu_{s,\text{top}}^{\prime }=\Delta \mu_{s,\mathrm{bot}}^{\prime }=0$, as hemodynamics should mostly result in absorption changes, and the baseline absorption may be considered homogeneous, $\mu_{a,\text{top}}=\mu_{a,\mathrm{bot}}=\mu_{a,\mathrm{Eff}}$, as the specific values of baseline absorption of the two layers do not significantly impact the absorption changes obtained with the six data types, as shown in Fig. 5).

## 5 Conclusion

The objective of this work is to explore the feasibility of discriminating superficial and deep tissue hemodynamics by performing non-invasive optical measurements with rich temporal and spatial information content. FD-NIRS is used to collect the amplitude [or intensity (I)] and the phase (ϕ) of photon-density waves launched into the tissue by intensity-modulated illumination. Two source–detector separations are used to collect data (I and ϕ) that feature different depth sensitivities in tissue. A special, symmetrical arrangement of two sources and two detectors is used to perform self-calibrating measurements of baseline optical properties, and robust measurements of absorption changes that are largely insensitive to instrumental drifts and motion artifacts. This work shows that the data types (I and ϕ) and geometrical arrangements of data collection (SD, DS) result in a set of measurements with a strong dependence on the baseline effective optical properties and absorption changes in the two-layered medium. This can be the basis for discriminating absorption dynamics in superficial and deep tissue by collecting data that are analyzed under the assumption of tissue homogeneity and by fitting them using a two-layered tissue model. In the case of muscle measurements, the thickness of the top layer (adipose tissue) can be measured with a skinfold caliper or ultrasound imaging. It is also possible to further expand the data space beyond what was done in this work, by introducing multiple DS sets of sources and detectors that feature multiple pairs of source–detector distances.

To the best of our knowledge, this is the first time that DS FD-NIRS is applied to muscle studies and that the information content of SD and DS FD-NIRS data is investigated in relation to the optical properties of two-layered media.

The approach proposed here, where measurement of effective optical properties and effective absorption changes are analyzed using diffusion theory for two-layered media, is an alternative to methods of data analysis with two-layered tissue models, as already proposed for studies in muscle^8^^,^^14^^,^^26^ and brain.^41^^–^^43^ These latter methods, however, may be less robust and require a greater data space (in the temporal and spatial domains) than the one proposed here. Future research will explore practical implementations of the method proposed in this work in comparison with a full-fledged two-layer tissue model for the hemodynamic characterization of layered tissue. This is a significant objective for non-invasive optical studies of biological tissue, with direct implications for skeletal muscle studies as well as human brain studies.

## Data Availability

Code and data utilized in this work are available from the authors upon reasonable request.
